# Toxocariasis and Epilepsy: Systematic Review and Meta-Analysis

**DOI:** 10.1371/journal.pntd.0001775

**Published:** 2012-08-14

**Authors:** Graziella Quattrocchi, Alessandra Nicoletti, Benoit Marin, Elisa Bruno, Michel Druet-Cabanac, Pierre-Marie Preux

**Affiliations:** 1 Department GF Ingrassia, Section of Neurosciences, University of Catania, Catania, Italy; 2 INSERM U1094, Tropical Neuroepidemiology, Limoges, France; 3 University of Limoges, School of Medicine, Institute of Neuroepidemiology and Tropical Neurology, Limoges, France; 4 CNRS FR 3503 GEIST, Limoges, France; 5 CHU Limoges, France; Uniformed Services University, United States of America

## Abstract

**Objective:**

Human toxocariasis is a zoonotic infection caused by the larval stages of *Toxocara canis* (*T. canis*) and less frequently *Toxocara cati (T. cati)*. A relationship between toxocariasis and epilepsy has been hypothesized. We conducted a systematic review and a meta-analysis of available data to evaluate the strength of association between epilepsy and *Toxocara* spp. seropositivity and to propose some guidelines for future surveys.

**Data Sources:**

Electronic databases, the database from the Institute of Neuroepidemiology and Tropical Neurology of the University of Limoges (http://www-ient.unilim.fr/) and the reference lists of all relevant papers and books were screened up to October 2011.

**Methods:**

We performed a systematic review of literature on toxocariasis (the exposure) and epilepsy (the outcome). Two authors independently assessed eligibility and study quality and extracted data. A common odds ratio (OR) was estimated using a random-effects meta-analysis model of aggregated published data.

**Results:**

Seven case-control studies met the inclusion criteria, for a total of 1867 participants (850 cases and 1017 controls). The percentage of seropositivity (presence of anti-*Toxocara* spp. antibodies) was higher among people with epilepsy (PWE) in all the included studies even if the association between epilepsy and *Toxocara* spp. seropositivity was statistically significant in only 4 studies, with crude ORs ranging 2.04–2.85. Another study bordered statistical significance, while in 2 of the included studies no significant association was found. A significant (p<0.001) common OR of 1.92 [95% confidence interval (CI) 1.50–2.44] was estimated. Similar results were found when meta-analysis was restricted to the studies considering an exclusively juvenile population and to surveys using Western Blot as confirmatory or diagnostic serological assay.

**Conclusion:**

Our results support the existence of a positive association between *Toxocara* spp. seropositivity and epilepsy. Further studies, possibly including incident cases, should be performed to better investigate the relationship between toxocariasis and epilepsy.

## Author Summary

Human toxocariasis is an infection caused by the larval stage of the worms *Toxocara canis* and less frequently *Toxocara cati*, common parasites of domestic and peridomestic dogs and cats. It is a cosmopolitan infection, occurring whenever the man-soil-dog relationship is particularly close, especially in tropical countries, where the humid climate favours the survival of parasite eggs in the soil, and in rural settings, where the poor hygiene increases the probability of human infection. Epilepsy affects nowadays at least 65 million of people worldwide and is particularly common in tropical areas, probably because of the presence of cases caused by infectious diseases largely absent in industrialized countries. For several decades, researchers have investigated the possible association between toxocariasis and epilepsy. In this study we conducted a statistical analysis of all the data available on the relationship between these two conditions. The combined results of the 7 studies included indicate an association between the two diseases. Further studies are necessary to demonstrate a causal relationship (i.e. toxocariasis causes epilepsy). Considering that toxocariasis is a preventable and common disease, a better understanding of the relationship between toxocariasis and epilepsy may contribute to improving prevention of epilepsy worldwide.

## Introduction

Human toxocariasis is a parasitic zoonosis caused by the larval stages of the ascarids *Toxocara canis (T. canis)*, the common roundworm of dogs, and by the roundworm of cats, *Toxocara cati* (*T. cati*) [1]. The reported prevalence of soil contamination with *Toxocara* spp. eggs is variable between studies, going from a percentage of 6.6 to 87.1% [2]–[9]. Therefore toxocariasis is one of the most prevalent zoonotic helminth infections, occurring whenever the man–soil–dog relationship is particularly close. High seroprevalence rates of *Toxocara* spp. (presence of sera anti-*Toxocara* spp. antibodies) have been found in tropical countries, where the humid climate favours the survival of parasite eggs in the soil, and in rural settings, where the poor hygiene and the rare administration of anthelmintic treatments to dogs increases the probability of human infection [10]–[12]. Nevertheless, the reported seroprevalence in apparently healthy adults from urban areas of Western countries is of 2–5% [13], whit a wider range (2.4%–31.0% [14], [15]) when considering all the studies carried out in Europe, independently from age of participants and type of setting. Despite being the most prevalent human helminthic infection in some industrialized countries [16], toxocariasis remains relatively unknown to the public [17] and the true magnitude of the global burden of *Toxocara* spp.-associated human disease has still to be evaluated [18].

Humans are infected by the accidental ingestion of embryonated *Toxocara* spp. eggs present in contaminated soil or food, or by the ingestion of encapsulated larvae contained in the raw tissues of paratenic hosts, such as cows, sheep or chickens [1], [19]. The clinical manifestations of human toxocariasis vary from asymptomatic infection to severe organ injury, depending on the parasite load, the sites of larval migration and the host's inflammatory response [20]. Two severe clinical syndromes are classically recognised: visceral larva migrans (VLM), systemic disease caused by larval migration through major organs, and ocular larva migrans (OLM), in which the disease is limited to the eyes and the optic nerves. Two less severe syndromes have also been described: ‘covert toxocariasis’, seen mainly in children and characterized by fever, headache, behavioural and sleep disturbances, cough, anorexia, abdominal pain, hepatomegaly, nausea and vomiting, and ‘common toxocariasis’, seen predominantly in adults with weakness, pruritus, rash, difficult breathing and abdominal pain [20]. Clinical involvement of the central nervous system (CNS) in visceral larva migrans is thought to be rare, although in experimental animals the larvae frequently migrate to the brain [21]–[23]. The CNS migration may lead to a variety of neurological disorders such as meningo-encephalitis, myelitis, cerebral vasculitis, optic neuritis [23], [24] and probably cognitive [25] and behavioural [26] disorders.

Concerning epilepsy, early reports have suggested a high exposure rate to *Toxocara* spp. among people with epilepsy (PWE) [27], [28]. In particular, in 1966 Woodruff et al. [27] found that 7.5% of PWE had a positive skin reaction to an antigen prepared from adult *T. canis*, in contrast to 2.1% of apparently healthy persons. In addition, they noted a statistically significant association between contact with dogs and positive skin test to toxocaral antigen in PWE. Following these preliminary observations and prompted by the development of serodiagnostic tests with improved sensitivity and specificity, further studies have been carried out in different populations to investigate the possible association between *Toxocara* spp. seropositivity and epilepsy, suggesting that toxocariasis could play a role in the incidence of epilepsy in endemic areas [29]–[31].

Considering that toxocariasis is one of the most common helminthiasis worldwide and that it is a potentially preventable disease, a correct estimate of the association between toxocariasis and epilepsy is necessary.

We carried out a systematic literature revision and a meta-analysis to evaluate the possible association between human toxocariasis and epilepsy and to highlight some methodological points to be taken into account for the elaboration of future surveys.

## Methods

### Literature search

A systematic search without past time or language restriction was conducted to identify published and unpublished articles dealing with the association between toxocariasis and epilepsy. The following online databases were independently examined by two researchers (GQ and BM): MEDLINE, IngentaConnect, ScienceDirect (Elsevier), Refdoc (ex ArticleScience), Scopus, Highwire. In addition, the database from the Institute of Neuroepidemiology and Tropical Neurology of the University of Limoges (IENT): Virtual Library on African Neurology, BVNA (http://www-ient.unilim.fr/), which contains more than 9000 references of medical dissertations, theses and articles dealing with tropical neurology and parasitology, was examined. In MEDLINE combined text words and Medical Subject Headings (MeSH) terminology were used. The following search key words and Boolean operators were entered: “toxocariasis” AND “epilepsy” AND “epidemiology”. The term “toxocarosis” as an alternative to “toxocariasis” was also considered. The literature search was adapted for the other databases. Titles and available abstracts were scanned for relevance, identifying papers requiring further consideration. Reference lists of all available reviews, primary studies and books found were screened manually. When necessary, corresponding authors of relevant studies were contacted. Experts in the field were also contacted to find out other eventual non-published studies. The systematic search was realized up to October 2011.

### Study selection

Considering epilepsy as the outcome and toxocariasis as the exposure, all the studies meeting the following eligibility criteria were included:

1. Presence of a control group (people without epilepsy, PWOE);
2. Information about methods used to assess epilepsy (i.e. survey, clinical examination, EEG, health records);
3. Serological or histopathological detection of toxocariasis;
4. Information about methods and criteria used for case-finding and control selection;
5. Possibility to determine the sample size of each of the following four groups in aggregated data: people with epilepsy seropositive for toxocariasis (PWE *Toxocara* spp.+), people with epilepsy not seropositive for toxocariasis (PWE *Toxocara* spp.−), people without epilepsy seropositive for toxocariasis (PWOE *Toxocara* spp.+), people without epilepsy not seropositive for toxocariasis (PWOE *Toxocara* spp.−).

Studies including only acute symptomatic seizures or specific seizure patterns or epileptic syndromes were excluded.

### Data extraction

Full copies of all reports identified by the electronic or hand searching were obtained and two reviewers (GQ and BM) independently assessed their eligibility and extracted data.

The following data were independently recorded in an *ad hoc* created collecting form: author, country, study design, study population (number, age group, gender, setting) and recruitment methods. For toxocariasis, specific information was recorded on methods used for diagnosis. Considering epilepsy, details on definition and assessment were extracted. Discrepancies between reviewers were rechecked and consensus was achieved by discussion.

For each survey, the crude odds ratio (OR) on the association between toxocariasis and epilepsy and the relative 95% confidence interval (CI) were recalculated. Furthermore, statistical power was calculated as *a priori* and *a posteriori*. A priori statistical powers were calculated following the hypothesis that the objective of the survey was to identify a minimum OR of 2 (i.e., *Toxocara* spp. exposure leads to twice more epilepsy) with one control per case, based on the number of PWE and the percentage of *Toxocara* spp. seropositivity in PWOE. The a posteriori statistical powers were calculated upon the results of the surveys. In both cases a 5% alpha risk was considered. Powers were calculated using Epi-Info 6.04 [32].

### Meta-analysis

To estimate the association between toxocariasis and epilepsy we performed a meta-analysis applying a random effects model, assuming that the true effect size of exposure varies from one study to the other, and that the studies in our analysis represent a random sample of effects that could have been observed [33]. A common risk was estimated as a common OR from all the studies. The homogeneity was tested by the Cochran Q test of heterogeneity. In order to account for the different age groups considered, the analysis was then separately applied to the studies including an exclusively juvenile population [30], [31]. Furthermore, considering that Western Blot (WB) is as sensitive but more specific than enzyme-linked immunosorbent assay (ELISA) [34], we also conducted an analysis restricted to the studies using WB as diagnostic or confirmatory test [35]–[38].

The meta-analysis was performed using EasyMA, 2001 version [39]. The PRISMA (Preferred Reporting Items for Systematic reviews and Meta-Analyses) statement [40] was used as a guide in the reporting of this study.

## Results

### Literature search

**Figure 1:**
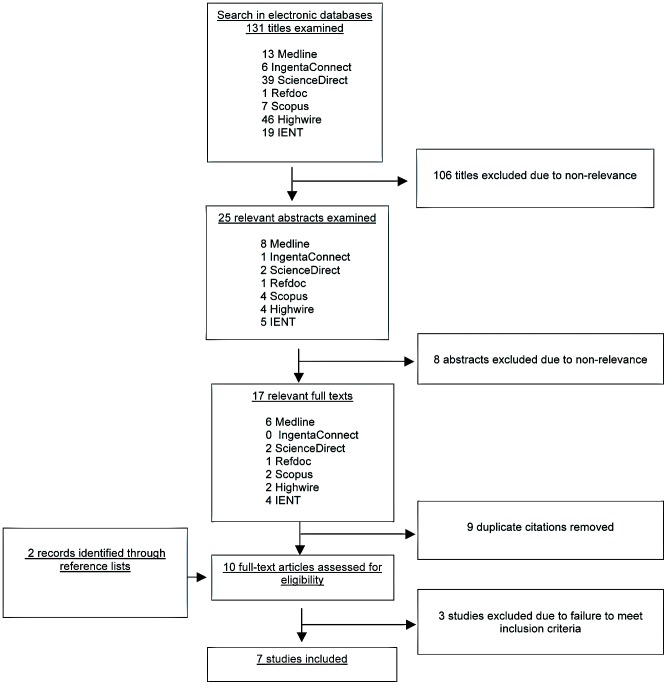
Flow chart of the literature search on the association between toxocariasis and epilepsy. IENT, Institute of Neuroepidemiology and Tropical Neurology of Limoges.

A flowchart summary of the literature search is shown in Figure 1. A PRISMA flowchart is also shown (Figure S1). Electronic search produced 131 publications, among which 25 dealt with epilepsy and toxocariasis. The removal of duplicate citations and the screening of abstracts permitted to isolate 8 documents [27], [31], [35]–[38], [41], [42]. Two additional publications [28], [30] were found by hand searching (reference lists check). Full text review of the 10 documents permitted to exclude 3 of them for not fulfilling the inclusion criteria: one [27] was excluded because methods to assess epilepsy were not reported and toxocariasis infection was detected through a skin test; furthermore the included cases consisted of a highly selected group of severe patients with epilepsy. Another study [28] was excluded because toxocariasis infection was exclusively assessed in a sample of PWE without control group. The last study [42] was excluded because of the lack of reporting of aggregated data for each group.

Considering the 7 articles meeting the inclusion criteria, the materials and methods of the study reported by Nicoletti et al. (2007) [36] had been previously detailed in Nsengiyumva et al. [43], while the study population of Winkler et al. (2008) [38] has been better described in Winkler et al. (2009) [44]. The methodological aspects of these articles have been therefore assessed considering both the publications.

### Included studies

Seven case-control studies [30], [31], [35]–[38], [41] were included, providing a total subjects number of 1867 (850 PWE and 1017 PWOE). Two of them [30], [31] considered a population aged 1–17 years while one excluded children aged 10 years or younger [38]. The studies were carried out in 6 different countries (USA, Italy, Bolivia, Turkey, Burundi and Tanzania), both in rural [35], [36], [38] and urban [30], [31], [37], [41] settings. In the study by Akyol et al. [41] 10% of participants were from rural areas, but no significant relationship was found between residency and seropositivity rate. The general characteristics of the included studies are shown in table 1.

**Table 1:** Description of the included studies looking for an association between toxocariasis and epilepsy.

			PWE Ascertainment			PWOE		Exposure
References	Country	Study design	Sources	Epilepsy definition and classification	Confirmation	Sources	Matching Criteria	Examinations
Glickman et al., J Pediatr 1979 [30]	USA	Case-control	Pediatric Hospital	Alter et al., 1972	Cases known by hospital	Outpatients or hospitalized controls	None	Sera Ab-ELISA
Arpino et al., Epilepsia 1990 [31]	Italy	Case-control	Pediatric Hospital	Not specified (“positive seizure history”)	Cases known by hospital	Pediatric Hospital	None	Sera Ab-ELISA
Nicoletti et al., Neurology 2002 [35]	Bolivia	Case-control	General population	ILAE 1993; ILAE 1981	Neurologist	General population	Sex, age ±5 years, same Community	Sera Ab-ELISA, WB
Akyol et al., Seizure 2007 [41]	Turkey	Case-control	Hospital, consecutively enrolled	Not specified (“idiopathic epilepsy”); ILAE 1981	Cases known by hospital	Volunteers, source not specified	None	Sera Ab-ELISA
Nicoletti et al., Epilepsia 2007 [36]	Burundi	Case-control	PWE identified by local health centers	ILAE 1993; ILAE 1981	Neurologist	Controls coming to hospital for vaccination or PWE neighbors	Age ±5 years, no blood relationship, same province	Sera WB
Nicoletti et al., Epilepsia 2008 [37]	Italy	Case-control	Epilepsy center, randomly selected	ILAE 1993; ILAE 1981	Neurologist	Subjects who went to hospital for hematological check, consecutively enrolled	Group-matched by age	Sera WB
Winkler et al., Trans R Soc Trop Med Hyg. 2008 [38]	Tanzania	Case-control	Hospital, age >10 years	WHO (1993) Winkler et al., 2007	Neurologist	Subjects who underwent CT for reasons other than seizures	None	Sera Ab-ELISA, WB, CSF Ab-ELISA

Ab-ELISA, antibodies enzyme-linked immunosorbent assay; CT, computerized tomography; CSF, cerebrospinal fluid; ILAE, International League Against Epilepsy; PWE, people with epilepsy; PWOE, people without epilepsy; WB, Western Blot; WHO, World Health Organization.

Three surveys had a matched case-control design [35]–[37] among them age was the only common matching criteria. Only one study was a population-based survey [35].

The epilepsy definition proposed by the International League Against Epilepsy (ILAE) in 1993 [45] was applied in 3 studies [35]–[37] while Glickman et al. [30] considered the definition proposed in 1972 by Alter [46], and Winkler et al. [38] defined epilepsy according to the World Health Organization (WHO) Neurosciences Research Protocol proposal [47]. In the work by Arpino et al. [31] a general definition of “positive seizure history” was considered as cases entry criteria. Considering seizures, 4 studies [35]–[37], [41] applied the classification of epilepsies and epileptic syndromes proposed by the ILAE in 1981 [48], while one [38] used an adjusted classification for rural African hospitals suggested in 2007 [49].

All PWE were prevalent cases and none of the studies clearly specified if active or lifetime epilepsy was considered, the second being more probable.

Controls were out- or in-patients attending the same hospital of cases [30] or people going to hospital for vaccination or haematological check [36], [37] or volunteers [41]. A negative history for seizures [31], [36]–[38], [41] and for both seizures and other neurological diseases [31], [36], [37] was considered for controls definition. In the population-based survey controls were selected from the same community, but different households, of cases [35], whereas another study selected controls from the same province of PWE excluding blood relationship [36]. In an attempt to determine the accuracy of the seizures classification EEG recordings were examined in some studies [31], [35]–[37].

A neurologist confirmed both cases and controls through anamnesis and complete neurological examination in 4 studies [35]–[38].

In order to obtain demographic data and information concerning factors possibly associated with *Toxocara* spp. exposure a questionnaire was administered to cases and control subjects in 5 studies [30], [31], [36], [37], [41]. Data were usually obtained by the patient's mother when the study population was infantile [30], [31]. The questionnaire version used was specified only in one study [36] and interviewers qualifications were stated only in 2 surveys [36], [37].

Presence of anti-*Toxocara* spp. antibodies in sera was assessed using antibodies-ELISA (Ab-ELISA, commercial or in-house kits) [30], [31], [41], or immunoblot [36], [37] or Ab-ELISA followed by WB confirmation [35], [38]. Laboratories performing the analysis were blind to the case-control status of sera samples in 3 studies [35]–[37].

### Association between toxocariasis and epilepsy

The results of the included studies are shown in Table 2. *Toxocara* spp. seropositivity ranged from 6.5% to 50.8% in the control group and from 12.0% to 59.7% in PWE. Seroprevalence rate was higher among PWE than control subjects in all the 7 included surveys, even if the association between *Toxocara* spp. seropositivity and epilepsy was statistically significant in 4 of them [30], [31], [35], [37]. In one study the crude OR bordered on statistical significance, anyway, after adjustments on other variables according to a multivariate model using the conditional logistic regression, a stronger and significant association was found [36].

**Table 2:** Results of the included case-control studies on the association between toxocariasis and epilepsy.

References	PWE, n	PWOE, n	Seropositivity in PWE, n (%)	Seropositivity in PWOE, n (%)	A priori statistical power, %*	A posteriori statistical power, %	OR (95% CI)	p-value
Glickman et al., J Pediatr 1979 [30]	84	108	19 (22.6)	11 (10.2)	33.6	65.3	2.58 (1.15–5.77)	0.018
Arpino et al., Epilepsia 1990 [31]	91	214	20 (21.9)	26 (12.1)	40.1	59.3	2.04 (1.07–3.88)	0.028
Nicoletti et al., Neurology 2002 [35]	113	233	28 (24.8)	28 (12.0)	47.6	85.0	2.41 (1.35–4.31)	0.002
Akyol et al., Seizure 2007 [41]	100	50	12 (12.0)	4 (8.0)	32.8	11.3	1.57 (0.48–5.14)	0.4
Nicoletti et al., Epilepsia 2007 [36]	191	191	114 (59.7)	97 (50.8)	90.9	41.0	1.43 (0.96–2.15)	0.08
Nicoletti et al., Epilepsia 2008 [37]	231	201	38 (16.4)	13 (6.5)	55.8	89.6	2.85 (1.47–5.51)	0.001
Winkler et al., Trans R Soc Trop Med Hyg. 2008 [38]	40	20	19 (47.5)	8 (40.0)	33.5	8.0	1.36 (0.46–4.03)	0.58

* Statistical power assuming an odds ratio equal to 2 with a type I error equal to 5% and one control per case.

OR, crude odds ratio; PWE, people with epilepsy as cases; PWOE, people without epilepsy as controls; seropositivity, presence of antibodies anti-*Toxocara* spp.; 95% CI, 95% confidence interval.

Significant crude ORs ranged between 2.04 and 2.85. A priori statistical power ranged 32.8–90.9% and a posteriori statistical power 8.0–89.6%.

### Meta-analysis

**Figure 2:**
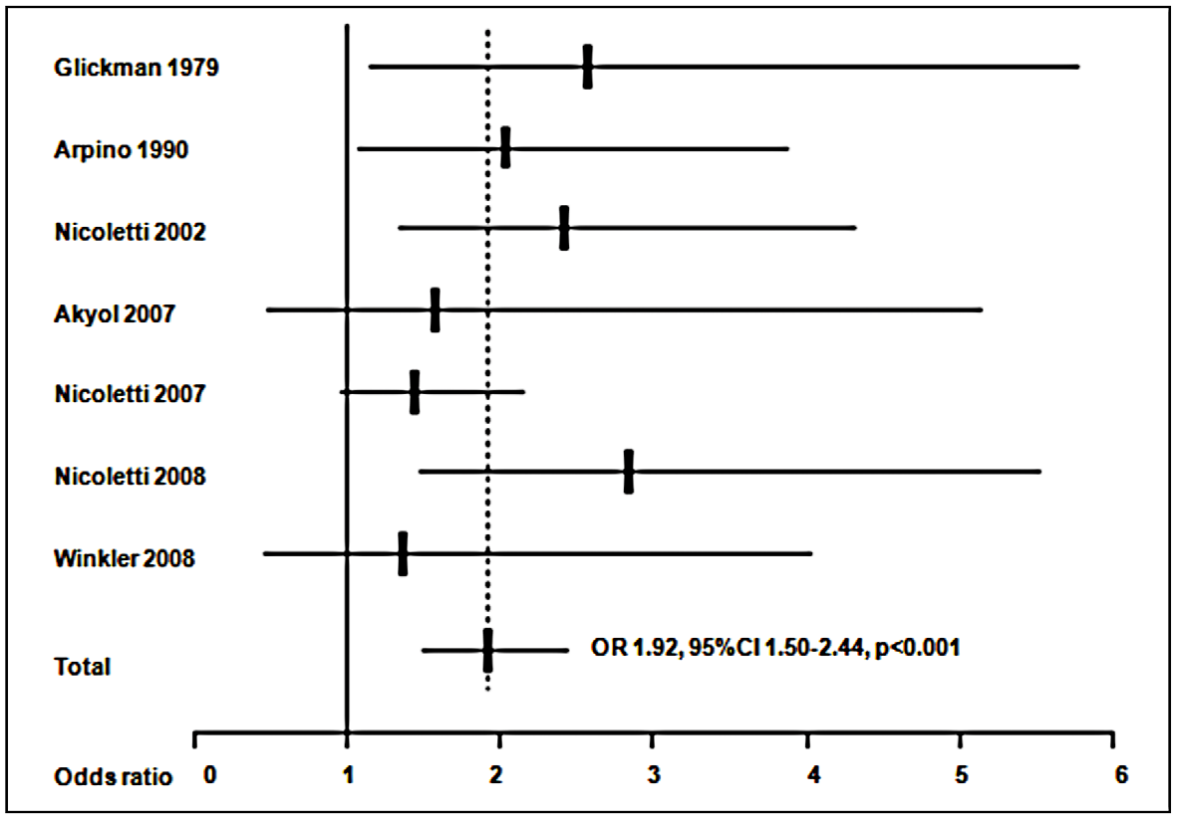
Meta-analysis of studies on the association between toxocariasis and epilepsy. ORs (Odds ratios) from each study and common OR estimated using a random effects model.

**Figure 3:**
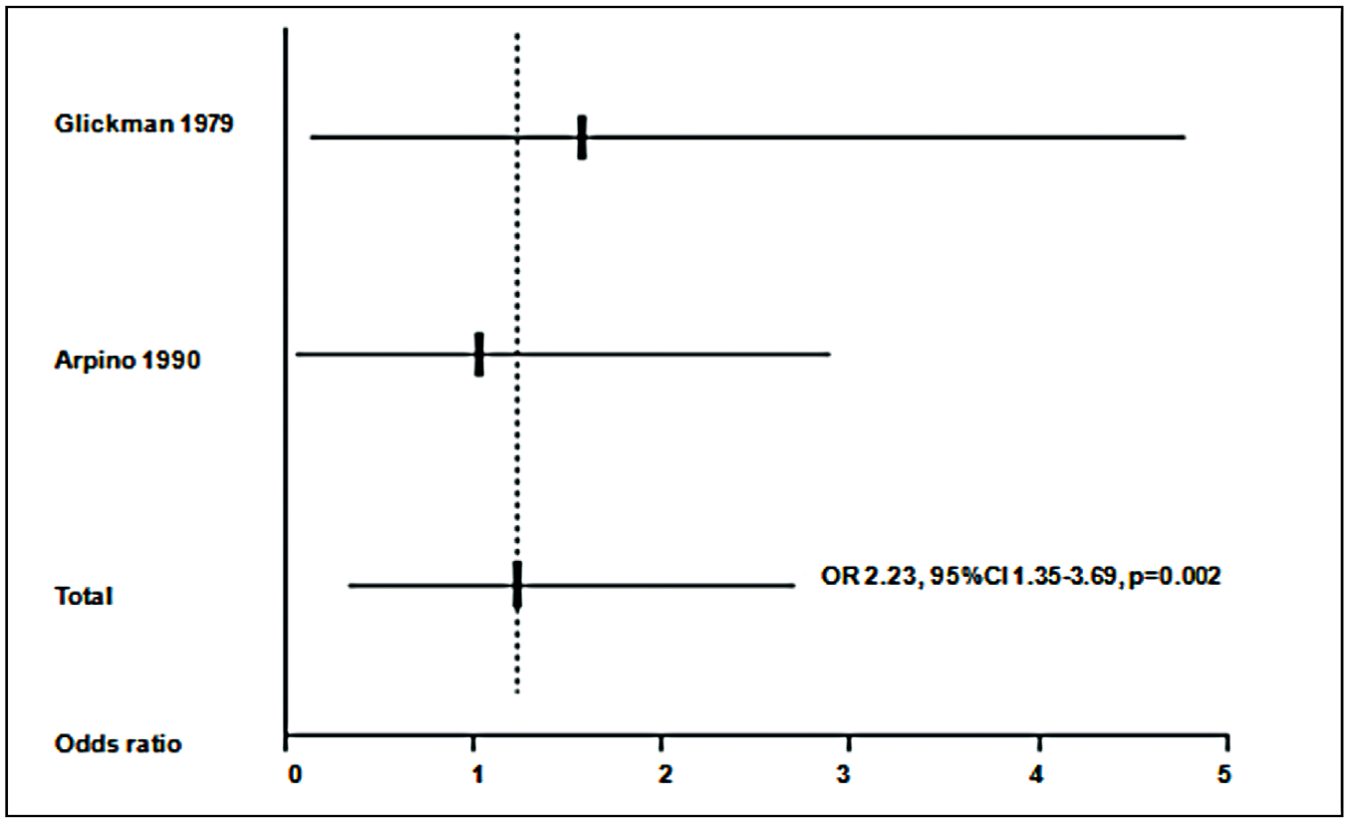
Meta-analysis of studies on toxocariasis and epilepsy including a young population. Age range: 1–17 years. ORs (Odds ratios) from each study and common OR estimated using a random effects model.

**Figure 4:**
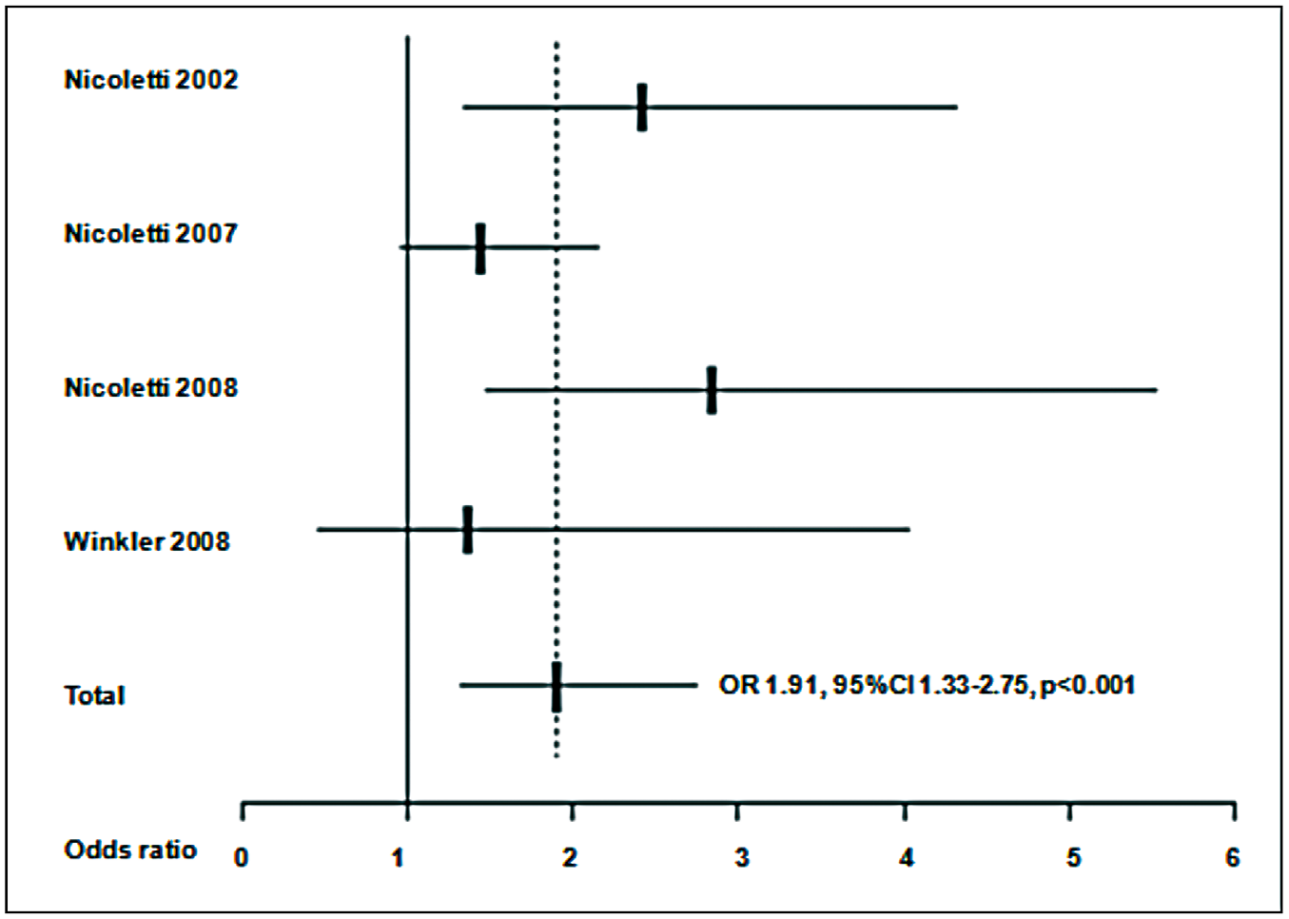
Meta-analysis of studies on toxocariasis and epilepsy using Western Blot as diagnostic or confirmatory test. ORs (Odds ratios) from each study and common OR estimated using a random effects model.

A meta-analysis was at first performed on all the 7 studies included. Results are presented in figure 2. A significant (p<0.001) common OR of 1.92 (95%CI 1.50–2.44) was estimated. The test of heterogeneity was not significant (p = 0.545), indicating homogeneity of the studies included. When analysis was restricted to the 2 studies considering only a juvenile population [30], [31], as shown in figure 3, a common OR of 2.23 (95% CI 1.35–3.69; p = 0.002) was found. The test for heterogeneity was also not significant (p = 0.655). The meta-analysis was at last restricted to the 4 studies using WB test [35]–[38], as shown in figure 4, leading to an OR of 1.91 (95% CI 1.33–2.75, p<0.001) and a non significant test for heterogeneity (p = 0.430).

## Discussion

We performed a systematic literature revision and a meta-analysis of available data to evaluate the association between epilepsy and toxocariasis. To our knowledge this is the first meta-analysis on this argument. Based on our literature search, we analyzed data from 7 case-control studies carried on in rural or urban settings and in various countries worldwide. We are confident that our literature search is exhaustive as conducted on several electronic databases and also on a specific database containing literature on tropical neurology and parasitology including theses and memos unpublished in international or electronic databases.

Seroprevalence rate of anti-*Toxocara* spp. antibodies was higher among PWE than control subjects in all the 7 studies analysed [30], [31], [35]–[38], [41] even if only 4 showed a significant positive association between *Toxocara* spp. seropositivity and epilepsy [30], [31], [35], [37] and a fifth reached statistical significance only after adjustment for other variables [36]. In our meta-analysis we found evidence of positive association, with a common OR of 1.92 (95%CI 1.50–2.44) and a lack of heterogeneity between the studies.

Our result is noteworthy for coming from studies across different populations in disparate geographic locations and socioeconomic climates. This consistency of observations among different populations is in favor for a causal relationship between toxocariasis and epilepsy [50]. Anyway, various important points should be taken into account when interpreting our data.

First of all, the studies evaluated were retrospective case-controls ascertaining both *Toxocara* spp. seropositivity and epilepsy in a cross-sectional manner; thus, the inclusion of “prevalent” rather than “incident” cases does not permit to demonstrate a temporal relationship between the exposure (*Toxocara* spp.) and the outcome (epilepsy) and doesn't allow to exclude a possible “reverse causality”. It has been in fact hypothesized that the abnormal behavior patterns (e.g. pica and hyperactivity) and the elevated number of falls to the ground of PWE (especially children or mentally retarded) could predispose them to *Toxocara* spp. exposure rather than the contrary [30]. In particular, evidence of association has been reported between *Toxocara* spp. seropositivity and mental retardation [51], [52]. We underline anyway that the study by Nicoletti et al. (2002) [35] found no statistical difference in seroprevalence between PWE with or without mental retardation. On the other hand, a significant difference in the frequency of mental retardation between seropositive and seronegative subjects was found by Nicoletti et al. (2008) [37], but it lead to only a slight reduction of association after restriction of the analysis to the PWE without mental retardation.

Selection of cases and controls represents one of the most important pitfalls in case-control studies. In the studies evaluated, with the exception of the only population based survey [35], PWE and PWOE were generally enrolled from a hospital setting, and their source population was often not clearly defined. This constitutes a possible recruitment bias, especially in rural settings, where people receiving care are not representative of the general population. In particular, concerning controls, hospital controls might resemble cases more than population controls, biasing OR towards the null [53]. Furthermore, a volunteer bias could have affected the study by Akyol et al. [41], where the control group was composed by volunteers coming from an undefined source. However, the population based survey showed a positive association, similar to the results found by the positive hospital based studies, suggesting that the selection bias effect could be limited.

Considering cases and controls characteristics, PWE and PWOE should be comparable at least for age, because the prevalence of both epilepsy and anti-*Toxocara* spp. antibodies vary with age, and for geographical provenience and education, which are likely related to the level of exposure to *Toxocara* spp. On this point, the studies examined are often lacking of detailed descriptive data. We report as an example, the comment by Quet et al. [54] on the study by Akyol et al. [41], which noticed how the greater number of students observed in the control group could suggest a higher education in controls than cases. The educational level was in fact expressed as a binary variable (less or more than primary school) in this study, which could give an unreliable estimation of education; in such cases the number of school years might be a more precise measure. In order to account for the different age groups included, and considering that young age seems to contribute to *Toxocara* spp. exposure [1], we restricted our meta-analysis to the studies with an exclusively juvenile population and we obtained also in this case a significant positive association (OR 2.23, 95% CI 1.35–3.69, p = 0.002). In particular, in the study by Arpino et al. [31] the relationship was more remarkable in children under 5 years old. Based on these findings, it has been suggested that the parasite may act as a cofactor in determining the occurrence of seizures in children with a predisposing background [31]. Only a prospective cohort follow-up study could avoid these biases. However such a design, leaving subjects exposed to toxocariasis and without intervention, is ethically not acceptable.

A potential weakness of our study is the use of different and not always clears epilepsy definitions in the included articles. On this point, considering that the lag time between *Toxocara* spp. infection and epilepsy occurrence is not yet defined, we underline the importance of including lifetime and not only active epilepsy, as likely properly done in the studies examined. On the other hand, the lack of exhaustive descriptive data on the age of onset, on the probable etiology and on seizures classification didn't permit us to differentiate our analysis for these factors. The significant positive association found in some studies between *Toxocara* spp. seropositivity and partial epilepsy could in fact be biologically plausible, given the higher prevalence of idiopathic epilepsy among the generalized forms [35], [37]; while the lack of association between partial epilepsy and toxocariasis found by Nicoletti et al. (2007) [36] has been related by the authors to a lack of power. In the study by Akyol et al. [41], besides the lack of a precise definition of epilepsy, the authors reported a higher frequency of generalized epilepsies, as expected, because of the inclusion of only cryptogenic (in the abstract) or idiopathic (in the methods) epilepsies [54]. This could have affected the results, showing no statistically significant association between toxocariasis and epilepsy. A correct classification of seizures, possibly with the help of EEG recordings, is therefore an important element that should be taken into account in future studies to permit a correct interpretation of the results.

Regarding the diagnosis of toxocariasis, the major limitation in confronting different studies consists in the heterogeneity of techniques (Ab-ELISA or WB or both) used to detect sera anti-*Toxocara* spp. antibodies, mostly due to different cost and availability. Also when considering ELISA, different kits (commercial or in house) with different sera dilutions were utilized and a serum pre-adsorption with larval Ascaris extracts was carried on only in some studies. It would have been interesting evaluating and reporting the sensitivity and specificity of the used assays, which has never been done in the studies examined. Considering that the WB confirmation of positive results from the ELISA (especially where pre-absorption is not carried out) has been recommended [55], and given the higher specificity of WB [34], we restricted our meta-analysis to the studies applying WB, obtaining results similar to the global analysis (OR 1.91, 95%CI 1.33–2.75, p<0.001).

When interpreting these data, we are of course aware that other factors, such as *Toxocara* spp. excretory-secretory (TES) antigen preparations, parasite strains, and WB technical procedures, could have influenced the results obtained by different investigators. It should also be kept in mind that a single seropositivity has limited pathological significance and could probably represent past rather than recent infection. Furthermore, the presence of sera antibodies against *Toxocara* spp. does not provide evidence of either an active systemic infection or a CNS involvement. Diagnosis of neurotoxocariasis is in fact based on the history; blood tests, including differential blood cell count and determination of serum total IgE; CSF investigation, including the detection of anti-*Toxocara* spp. antibodies and neuroimaging [13].

The absence of significant results was associated with a lower power (type II error), making not really surprising the lack of statistical confirmation of the difference found. The statistical power of a study can be improved performing surveys in areas with high level of exposure assessed with the most sensitive assay or, when the number of cases is small, increasing the ratio of controls to cases up to 4/1 [53]. The low a posteriori power of the studies by Winkler et al. [38] (8.0%) and Akyol et al. [41] (11.3%) could be mostly accounted to the small sample size and in particular the lower number of controls than cases, highlighting one more time the central role of the elaboration of the control group.

In our paper we referred to toxocariasis etiological agent as *Toxocara* spp. and not only *T. canis*. TES in fact are not species-specific and the differentiation between *T. canis* and *T. cati* remains challenging. Considering the prominence historically given to *T. canis*, the role of *T. cati* in human toxocariasis could have been underestimated. Further work should be encouraged to differentiate the two parasites and to better address future prevention strategies [56].

The most frequent infectious agent involved in the differential diagnosis of subjects with late-onset epilepsy or inflammatory brain nodules is the larval stage of *Taenia solium (T. solium)*, aetiological agent of neurocysticercosis (NCC). Concomitant *T. solium* and *Toxocara* spp. seropositivity is a possible event in areas endemic for both helminthes. Anyway, albeit there is yet no evidence on the mechanisms underlying toxocariasis-induced epilepsy, according to us toxocariasis should not be ruled out as an accidental association. First of all, the presence of anti-*T. solium* antibodies, as in the case of toxocariasis, could represent only a previous exposure without established infection. Furthermore, considering the studies included in our analysis, in the study by Nicoletti et al. (2002) [35] only 7 PWE over a total of 113 were positive to both *T. solium* and *Toxocara* spp. and in the study by Nicoletti et al. (2007) [36], finding a positive association between *Toxocara* spp. seropositivity and epilepsy, seropositivity for cysticercosis was considered as a variable in the multivarate analysis. Of course, the interpretation of serological results should always take into account the background seroprevalence of both *Toxocara* spp. and *T. solium* in the studied population and cysticercosis seropositivity should always be evaluated as a possible confounder when carrying on surveys on infectious agents and epilepsy.

In conclusion, a positive association between *Toxocara* spp.-seropositivity and epilepsy could be hypothesized; nevertheless, even the modestly strong association demonstrated in our meta-analysis does not necessarily prove causality (i.e., *Toxocara* spp. infestation caused the epilepsy). Further studies, considering incident rather than prevalent cases and with a population-based design, should be performed. An internationally accepted epilepsy definition and seizures classification should be applied and cases and controls should be comparable at least for age, sex, geographic provenience, education and socio-economic background. Pica, pet owning, mental retardation and other possible toxocariasis risk factors should be assessed trough a validated questionnaire administered by trained investigators and assessors and laboratory personnel should be blind to the status of participants. The improvement of techniques permitting to distinguish recent from past infections, such as antigen-ELISA (Ag-ELISA), should be encouraged in order to better investigate the time relationship between *Toxocara* spp. infection and epilepsy occurrence.

Assessing the link between toxocariasis and epilepsy is of interest as toxocariasis is a potentially preventable disease. Nowadays, despite the implementation of regular and intensive de-worming programs in western countries, the parasite still prevails, indicating that prevention is not easy in practice. Good hygiene practices should be encouraged and further strategies to prevent *Toxocara* spp. transmission should be identified and applied, permitting to experimentally investigate the causation hypothesis [50]. The existence of a causal relationship and the estimation of the impact of toxocariasis on the global burden of epilepsy may strongly contribute in encouraging further programs on toxocariasis prevention worldwide, in order to control both the *Toxocara* spp. transmission and the related epilepsy burden.

## Supporting Information

Checklist S1 **Prisma checklist.**
(DOC)

Figure S1 **Prisma Flow Diagram of the literature search on the association between toxocariasis and epilepsy.**
(DOC)
