# Supplementary material for: Toxocariasis and Epilepsy: Systematic Review and Meta-Analysis
Source: PLoS Negl Trop Dis. 2012 Aug 14;6(8):e1775. doi: 10.1371/journal.pntd.0001775 (PMC3419195; doi:10.1371/journal.pntd.0001775)
Supplement: Figure S1 — Prisma Flow Diagram of the literature search on the association between toxocariasis and epilepsy. (DOC) [file pntd.0001775.s002.doc]

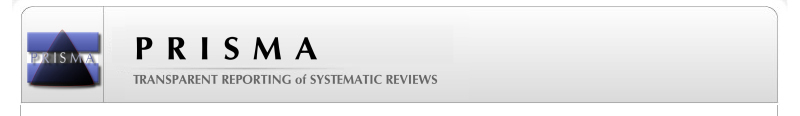
**PRISMA 2009 Flow Diagram**

**Screening**

**Included**

**Eligibility**

**Identification**

Records identified through database searching
(n = 131)

Additional records identified through other sources
(n = 2)

Relevant full-text articles

(n = 19)

Records excluded
(n = 114)

Full-text articles assessed for eligibility
(n = 10)

Full-text articles excluded, with reasons
(n = 3)

Studies included in qualitative synthesis
(n = 7)

Studies included in quantitative synthesis (meta-analysis)
(n = 7)

Records screened
(n = 133)

Duplicate full-text articles excluded
(n = 9)
